# Induction of a Protective Heterosubtypic Immune Response Against the Influenza Virus by using Recombinant Adenoviral Vectors Expressing Hemagglutinin of the Influenza H5 Virus

**Published:** 2010-04

**Authors:** M.M. Shmarov, E.S. Sedova, L.V. Verkhovskaya, I.A. Rudneva, E.A. Bogacheva, Yu.A. Barykova, D.N. Shcherbinin, A.A. Lysenko, I.L. Tutykhina, D.Y. Logunov, Yu.A. Smirnov, B.S. Naroditsky, A.L. Gintsburg

**Affiliations:** Gamaleya Research Institute of Epidemiology and Microbiology, Russian Academy of Medical Sciences; Ivanovsky Virology Research Institute, Russian Academy of Medical Sciences

**Keywords:** adenoviral vector, influenza virus, hemagglutinin, immunization, heterosubtypic protection

## Abstract

Abstract Influenza viruses are characterized by a high degree of antigenic variability, which causes the annual emergence of flu epidemics and irregularly timed pandemics caused by viruses with new antigenic and biological traits. Novel approaches to vaccination can help circumvent this problem. One of these new methods incorporates genetic vaccines based on adenoviral vectors. Recombinant adenoviral vectors which contain hemagglutinin–encoding genes from avian H5N1 and H5N2 (Ad–HA5–1 and Ad–HA5–2) influenza viruses were obtained using the AdEasy Adenoviral Vector System (Stratagene). Laboratory mice received a double intranasal vaccination with Ad–HA5–1 and Ad–HA5–2. This study demonstrates that immunization with recombinant adenoviruses bearing the Н 5 influenza virus hemagglutinin gene induces a immune response which protects immunized mice from a lethal dose of the H5 influenza virus. Moreover, it also protects the host from a lethal dose of the H1 virus, which belongs to the same clade as H5, but does not confer protection from the subtype H3 influenza virus, which belongs to a different clade.

## INTRODUCTION

Our data allow us to conclude that adenoviral vectors may become a universal platform for obtaining vaccines against seasonal and pandemic strains of the influenza virus.

Influenza A viruses can cause severe epidemics. These viruses are widespread in nature and can infect humans, many mammalian species (horses, pigs, seals, etc.), and all species of birds [6, 17]. Different strains of the influenza virus are traditionally named after the numerical indexes of their surface antigens (hemagglutinin (HA) and neuraminidase (NA)). Overall, there are 15 subtypes of hemagglutinin and 9 subtypes of neuraminidase with minimal cross–activity in serological reactions between subtypes [18]. Two subtypes of hemagglutinin ( Н 1, Н 3) and two types of neuraminidase (N1–N2) are currently circulating in the human population [18, 20]. Another potential threat to humans comes from avian influenza viruses, which have H5 hemagglutinin, since numerous cases of humans being infected by the H5N1 have been reported as far back as 1997. This virus was first detected in a human organism in China more than 10 years ago [7]; according to the WHO, the overall number of humans infected with H5N1 is 478, and 286 of such cases resulted in death. Influenza A viruses are characterized by a very high degree of antigen variability. The most variable entities are the surface glycoproteins of the viral particle (hemagglutinin and neuraminidase). Two mechanisms of variation are known. The first is antigenic drift. When a viral population is under pressure from the immune system, mutations that allow the virus to escape this controlling influence give the virus a serious advantage, and thus these mutations are conserved. This means that new antigenic variants of hemagglutinin and neuraminidase are constantly replacing each other. This creates the basis for epidemics, since immunity against the previous virus strain, even if it still belongs to the same subtype, is not sufficient to neutralize the new strain. Unfortunately the use of modern subunit and inactivated vaccines does not solve the problem, since they confer protection only from the strain which was used to obtain the vaccine. For this reason new vaccines for the protection of the population need to be created constantly.

The second mechanism of influenza virus variation is the antigenic shift, which is the alteration of the antigenic formula of a virus via the exchange of a gene (genes) and the corresponding protein (proteins). Antigenic shift is based on the reassortment or recombination of genes, which can take place if an organism is infected by two or more virus strains [19]. Shifts most often affect the antigenic structure of hemagglutinin, shifts in neuraminidase being less common. Thus, pandemic variants of the influenza virus with new antigenic and biological traits can emerge at irregular time periods, causing severe diseases and the death of a large number of people [6, 17]. For instance, the pandemic of the so–called “Spanish flu” – an H1N1 influenza virus in 1918–1920 – caused the death of around 50 million people worldwide. In June 2009, the World Health Organization (WHO) called the new H1N1 virus, cases of which started cropping up in April 2009, a 6th degree pandemic threat. This virus was the result of a reassortment, which combined the genes of avian, human, and porcine viruses [8]. If a new pandemic virus emerges, the creation of a vaccine takes too long, thus preventing a swift end to the spread of the dangerous virus. Circumventing this problem requires the creation of novel approaches to vaccinations against influenza viruses. One such approach is the creation of genetic vaccines based on viral vectors [5, 11]. The most promising type of genetic vaccines is a vaccine based on adenoviral vectors [1, 2]. Until recently it was thought that influenza–targeted vaccines, including genetic varieties which used variable surface antigens of influenza viruses (hemagglutinin and/or neuraminidase), were strictly specific to the strain whose antigen was used during the creation of the vaccine. However, studies performed in recent years (2008–2009) demonstrate that genetic vaccines based on adenoviruses which contain the hemagglutinin gene from an A–type influenza virus can induce cross–immunity both inside a single subtype of influenza virus [3] and between different subtypes of a virus that share the same subtype of hemagglutinin [15]. Such an effect may be caused by the fact that an organism vaccinated by an adenovirus receives an influenza virus hemagglutinin gene, which is then effectively expressed in the cell and is exposed on the plasma membrane, retaining its native ternary structure. This induces both cellular and humoral responses to the conservative epitopes of the influenza virus hemagglutinin. It was demonstrated that antibodies obtained from human plasma cells infected by an H5N1 influenza virus could also neutralize H1–subtype viruses as well [4]. Data published in 2008 also show that influenza viruses can be divided into two groups according to the presence of highly conservative conformational epitopes in the hemagglutinin molecule which can be identified by broad–range antibodies. The first group includes subtypes H1, H2, H5, H6, H8, H9, H11, H12, H13, and H16; the second includes subtypes H3, H4, H7, H10, H14 and H15. Antibodies against the antigenic determinants of the first group are not equally effective for the neutralization of viruses of this group, but they do not neutralize viruses from the second group [14]. These groups were divided into four subgroups or clades according to the presence of conservative epitopes: Н 1 clade (H1, H2, H5, H6, H11, H13 and H16 hemagglutinins), Н 9 clade (H9, Н 8 and Н 12), Н 7 clade ( Н 15, Н 7 and Н 10) and Н 3 clade ( Н 3, Н 14 and Н 4).

Thus we have proposed that vaccination by an adenoviral vector bearing the the hemagglutinin gene of an Influenza A virus can lead to the production of antibodies against conservative epitopes (some of which may be conformational) of the major Influenza A surface antigen. This provides a cross–subtype immune response not only against influenza A viruses from a single subtype, but also against viruses of various subtypes which belong to the same clade.

This study used recombinant human adenoviruses of the fifth serotype which bore hemagglutinin genes from the avian influenza viruses H5N2 and H5N1, since the Н 5 subtype avian viruses are likely candidates for the next pandemic strain. It was shown that immunization with recombinant adenoviruses induced the induction of a protective immune response, which allowed mice to survive lethal doses of its “native” H5 strain, and protected against a lethal dose of H1, which belongs to the same clade as H5. However this immune response did not protect the mice from an H3–subtype virus belonging to a different clade.

## EXPERIMENTAL PROCEDURES

**Viruses**. This study used an avian influenza virus A/ Mallard/Pennsylvania/10218/84 (H5N2) and human influenza viruses A/USSR/90/77 (H1N1) and A/Aichi/2/68 (H3N2) adapted for use on mice [9]. The viruses were accumulated in the alantoic fluid of chicken embryos at 37° С for 48 hours. The virus–containing alantoic fluid was stored at –70° С . The titer of the virus was calculated by titration in chicken embryos. The 50% lethal dosage (LD_50_) was calculated by titration on mice.

**Obtaining recombinant adenoviruses**. Plasmids and recombinant adenoviruses were obtained by using hemagglutinin genes from an avian influenza A/Mallard/Pennsylvania/10218/84 (H5N2) virus and a A/Duck/Novosibirsk/56/2005 (H5N1) ( Н 5–2 and Н 5–1, respectively). The cDNA of the viral genome of A/Mallard/Pennsylvania/10218/84 (H5N2) was obtained by reverse transcription using a Reverse Tpanscription System (Invitrogene, United States). The hemagglutinin Н A5–2 was obtained by amplifying the cDNA with primers which flanked the hemagglutinin gene. The Н 5–1 hemagglutinin gene was supplied by the Viral Disease diagnostic laboratory of the All–Russian Research Institute of Animal Health (Vladimir).

Recombinant adenoviruses Ad–HA5–1 and Ad–HA5–2 were obtained by homologous recombination in * E.coli * cells using the AdEasy Adenoviral Vector System (Stratagene). The obtained adenoviruses were purified and concentrated by double ultracentrifugation in a cesium chloride gradient. A recombinant serotype 5 human adenovirus with no expression cassette in the region of deletion in the E1 area of the genome was used as a control (Ad–null). The titers of the Ad–H А 5–1, Ad–H А 5–2 and Ad–null preparations were calculated using plaque formation in a HEK–293 cell culture.

**Detection of hemagglutinin gene expression by IFA**. Hemagglutinin gene expression by the recombinant viruses was performed on HEK–293 cells inoculated with the Ad–H А 5–1 and Ad–H А 5–2 vectors at a dosage of 100 particles per cell. After 24 h of incubation, the cells were lysed and ELISA was performed using a kit for the detection of H5 avian influenza strain hemagglutinin (BioAssay).

**Measuring levels of hemagglutinin–binding antibodies in murine serums**. Antibody levels in murine serums were assayed 21 days after the second immunization. The hemagglutinin–binding antibody level in murine serums was assayed by a hemagglutination–inhibition reaction performed according to the WHO/CDS/CR S/NC S/2002.5 protocol using chicken erythrocytes.

**Mice**. The mice used in this study were females of the BALB/c line and weighed about 7–9 g.

**Animal immunization**. The mice were divided into groups (ten animals per group) and immunized twice by an intranasal dose of recombinant Ad–H А 5–1 and Ad–H А 5–2 viruses, 10^8^ PFU/mouse. The interval between immunizations was 21 days. The control groups were immunized with the Ad–null virus or treated with PBS solution instead.

**Animal infection**. Twenty–one days after the second immunization, the mice were lightly anaesthetized with ether and then received a lethal dose (50 LD_50_) of the A/Mallard/Pennsylvania/ 10218/84 (H5N2) strain or lethal doses (10 LD_50_) of A/USSR/90/77 (H1N1) and A/Aichi/2/68 (H3N2) strains. The survival and changes in mouse weight were measured for 16 days after infection.

## RESULTS AND DISCUSSION

**Fig. 1:**
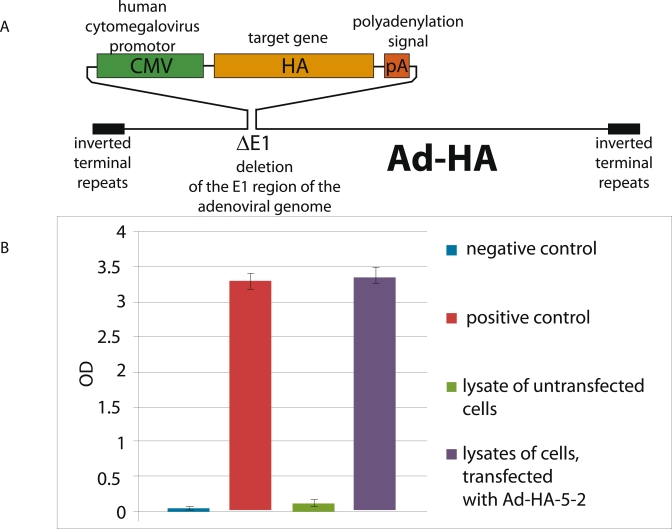
The recombinant human adenovirus of the 5th serotype expresses the hemagglutinin gene of the avian H5N2 influenza virus (A/Mallard/ Pensylvania/10218/84) in infected cells. (А) A schematic of the genome of the recombinant adenovirus, which bears the influenza virus hemag- glutinin. (B) Expression of the H5N2 virus hemagglutinin by line 293 human embryo kidney cells infected with Ad-HА5-2 (ELISA): (-) negative control, (-) positive control, (-) lysates prepared from cells untransfected by Ad- HA5-2, and (-) lysates prepared from cells transfected by Ad-HA5-2

**Detection of hemaglutinnin gene expression in recombinant Ad–H А  5–1 and Ad–H  А  5–2 adenoviruses. ** Schematics of the genomes of the recombinant Ad– НА 5–1 and Ad–HA5–2 adenoviruses, which were obtained by homologous recombination in * E.coli * cells, are presented in Fig. 1A. The obtained preparations of adenoviral vectors were assayed for the presence of avian H5N1 and H5N2 influenza hemagglutinin gene insertions in the genomic DNA by PCR (data not shown).

Lysates obtained from HEK–293 cells infected with the recombinant Ad–HA5–2 adenovirus were assayed for the expression of recombinant hemagglutinin by ELISA using a kit for the detection of avian H5 influenza hemagglutinin (Fig. 1B). The expression of avian H5N1 influenza hemagglutinin by the Ad–HA5–1 adenovirus was assayed similarly (data not shown).

**Fig. 2:**
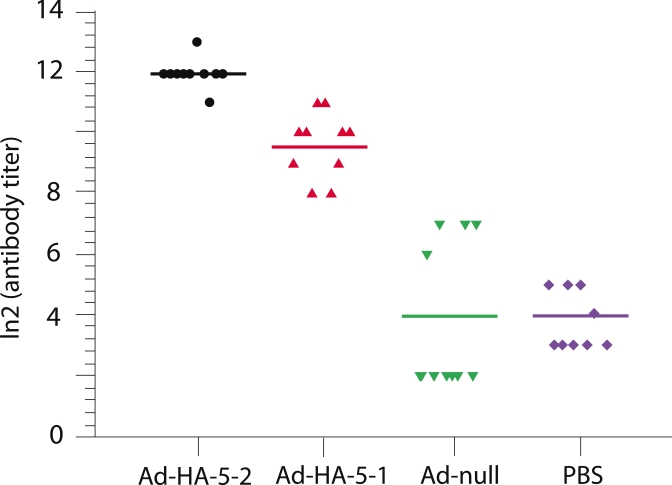
Level of specific hemagglutinating antibodies which neutralize the avian H5N2 influenza virus in sera obtained from mice immunized by the recombinant Ad-HA5-1 and Ad-HA5-2 adenoviruses. Mice were immunized with Ad-HA5-1 and Ad-HA5-2 adenoviruses twice. Mice immunized by either Ad-null or PBS solution were used as controls. Anti- body titers in mouse sera were assayed by HRR against the influenza A/ Mallard/Pensylvania/10218/84 (H5N2) virus. A significant difference was detected between the control groups immunized by Ad-null and PBS and the groups vaccinated by Ad-HA5-1 and Ad-HA5-2 (p < 0.05)

**Assay for the immunogenicity of Ad–HA5–2–expressed hemagglutinin and the cross–immunogenicity of Ad–HA5–1–expressed hemagglutinin**. The level of antibodies secreted against the recombinant hemagglutinin produced by the Ad–HA5–2 adenovirus was assayed using a hemagglutination–inhibition reaction. Mice of the BALB/c line were immunized with the Ad–HA5–2 adenovirus. Mice injected with PBS buffer were used as controls. Mice sera obtained 21 days after repeated immunization were analyzed for the presence of specific antibodies against avian influenza strain A/Mallard/Pennsylvania/10218/84 (H5N2). The hemagglutination–inhibition reaction results are presented in Fig. 2. The sera from mice immunized with the recombinant Ad–HA5–2 adenovirus appeared to have high levels of antibodies, which inhibited the agglutination of erythrocytes by the H5N2 virus. These data indicate the induction of a humoral immune response specific to the H5N2 avian influenza virus following the intranasal injection of a recombinant adenovirus expressing the H5 hemagglutinin gene.

The cross–immunogenicity of Ad–HA5–1–expressed hemagglutinin was assayed in a similar manner. The sera obtained after immunization were assayed for the presence of specific antibodies against avian influenza A/Mallard/Pennsylvania/10218/84 (H5N2) hemagglutinin using hemagglutination–inhibition reaction (Fig. 2.). The tested mice sera had high levels of antibodies that prevented the agglutination of erythrocytes by the H5N2 influenza virus.

These results indicated the induction of a heterosubtypic humoral immune response specific to the H5N2 avian influenza virus following the immunization of mice by adenoviruses expressing the hemagglutinin gene from the H5N1 avian influenza virus. The amount of antibodies specific to the H5N1 viral hemagglutinin was lower than in the case of immunization by an adenoviral vector bearing the H5N2 influenza hemagglutinin, but was still relatively high compared to the control group.

In control (PBS) sera, some nonspecific hemagglutination inhibition was observed due to the components of the murine serum.

**Fig. 3:**
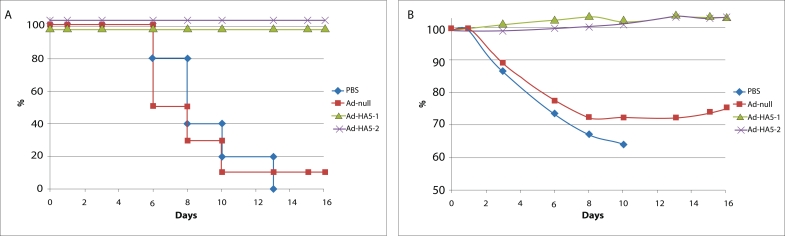
Analysis of the protective hemagglutinin-specific immune response in mice immunized by recombinant Ad-HA5-1 and Ad-HA5-2 adenoviruses and later infected by a lethal dose of avian influenza virus H5N2. (А) Survival of mice infected by a lethal dose (50LD50) of the A/Mallard/ Pensylvania/10218/84H5N2 (H5N2)-strain virus. Mice were immunized twice with the Ad-HA5-1 and Ad-HA5-2 adenoviruses. Control mice were immunized twice either with Ad-null or with PBC solution. The differences in survival between the Ad-HA5-2 (Ad-HA5-1) groups and the PBS control group (р < 0.00001) and the Ad-null control group (p < 0.0001) were statistically significant. (B) Changes in mouse weight as measured for the mice treated with 50LD50 of the A/Mallard/Pensylvania/10218/84H5N2 (H5N2) virus.

**Protective immunity of mice immunized by Ad–HA5–2 and cross–immunity of Ad–HA5–1–infected mice against H5N2 avian influenza virus infection**. In order to study the protective effect of a double immunization with Ad–HA5–2, the immunized animals were infected with a lethal dose (50 LD_50_) of the A/Mallard/ Pennsylvania/10218/84 (H5N2) influenza virus. The control mice were injected with the Ad–null recombinant adenovirus and with PBS solution. Mice which were immunized with the AdHA5–2 adenovirus did not lose any weight after infection and had 100% survival. The control PBS group, on the other hand, showed a 100% mortality rate over the course of 10 days and a 20% reduction in weight. The Ad–null group showed 20% survival and 30% weight reduction (Fig. 3). Thus, immunization with the recombinant Ad–H А 5–2 adenovirus protected mice from a lethal H5N2 influenza virus infection. In order to study the induction of protective cross–immunity, mice were immunized as was described above by a recombinant Ad–H А 5–1 adenovirus and infected with a lethal dose (50 LD_50_) of the A/Mallard/Pennsylvania/10218/84 (H5N2) virus. The immunization of mice with the Ad–H А 5–1 adenovirus induced a heterosubtypic cross–immune response against the H5N2 influenza virus. The immunized mice showed no reduction in weight and had a 100% survival score (Fig. 3).

**Fig. 4:**
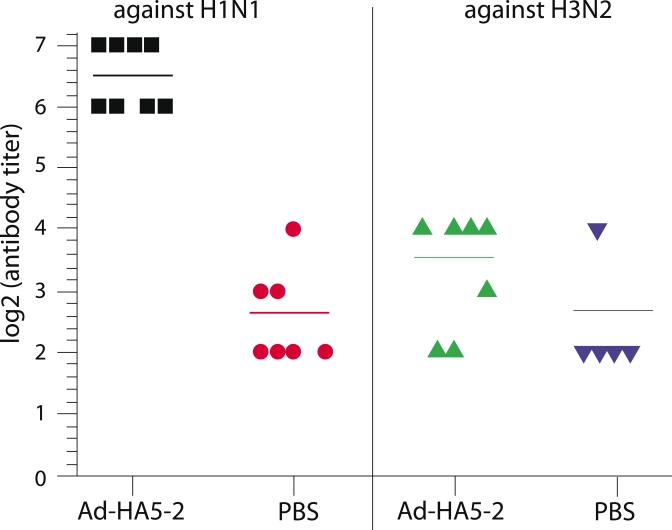
The level of specific hemagglutinating antibodies against H1N1 and H3N2 viruses in the sera of mice immunized with the recombinant Ad-HA5-2 adenovirus. The mice were immunized with the Ad-HA5-2 adenovirus twice. Control mice were immunized with PBS solution twice. The antibody titer in mice sera was assayed using HRR against A/ USSR/90/77 (H1N1) and A/Aichi/2/68 (H3N2) viruses. The difference between the vaccinated and control groups as assayed by HRR against the H1N1 was statistically significant (p = 0.05), while the HRR against the H3N2 virus showed no significant difference (p > 0.05).

**Assaying the cross–immunogenicity of Ad–HA5–2–expressed hemagglutinin against H1N1 and H3N2 viruses**. The cross–immunogenicity of recombinant hemagglutinin expressed by the Ad–HA5–2 adenovirus was assayed by the hemagglutination–inhibition reaction. BALB/c mice were immunized by the Ad–HA5–2 adenovirus as previously described. Control mice were intranasally injected with PBS solution. Samples of mice serum were collected 21 days after the second immunization. These samples were assayed for the presence of antibodies specific to A/USSR/90/77

(H1N1) and A/Aichi/2/68 (H3N2) influenza hemagglutinin (results show on Fig. 4). Sera from mice immunized with the recombinant Ad–HA5–2 adenovirus were found to exhibit a pronounced increase of anti–H1N1 antibody titers in a hemagglutination–inhibition reaction assay when compared to the control group. There was no statistically significant difference in the hemagglutination inhibition levels between the anti–H3N2 reaction in sera from the Ad–HA5–2–immunized mice and the nonspecific inhibition due to components of the control murine serum.

The obtained result indicated the induction of a humoral immune response specific to the H1N1 influenza virus in response to immunization by a recombinant Ad–HA5–2 adenovirus. The efficacy of the humoral immune response against the H3N2 influenza virus proved to be insignificant.

**Fig. 5:**
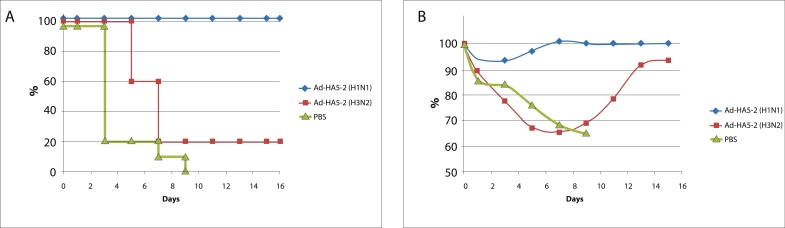
Analysis of the protective hemagglutinin-specific response in mice immunized by the recombinant Ad-HA5-2 adenovirus and later infected by a lethal dose of either the H1N1 or H3N2 virus. (А) Survival of mice infected with a lethal dose (10LD50) of A/USSR/90/77 (H1N1) or A/Aichi/2/68 (H3N2) influenza virus. Mice were immunized with the Ad-HA5-2 twice in concentration 108 PFU/mouse. Control mice were injected with PBS solution twice. The differences in survival between the Ad-HA5-2 group (infected with H1N1) and the PBS control group were statistically significant, while the differences between the survival in the Ad-HA5-2 group (infected with H3N2) and the PBS control group were not (p = 0.5). (B) Changes in the weight of mice infected with a lethal dose (10LD50) of A/USSR/90/77 (H1N1) or A/Aichi/2/68 (H3N2) virus

**Assaying the cross–protection of mice immunized with Ad–HA5–2 against the influenza A viruses H1N1 and H3N2**. After the second Ad–HA5–2 immunization, mice were injected with lethal doses (10 LD_50_) of A/USSR/90/77 (H1N1) and A/Aichi/2/68 (H3N2) viruses. Control mice were intranasally injected with PBS solution. This experiment showed that immunizing animals with the recombinant Ad–HA5–2 adenovirus protected mice from a lethal dose of the H1N1 influenza virus. Mice did not die during the whole observation period, and their weight reduction was approximately 5%. The control group injected with PBS showed 100% mortality over 9 days and a weight reduction of 30%. Immunizing mice with the Ad–HA5–2 adenovirus did not protect mice from a lethal dose of the H3N2 influenza virus. The immunized mice exhibited 20% survival and almost 40% weight reduction (Fig. 5).

**Discussion**. Protecting the human population from the constant threat of influenza is problematic due to the difficulty of predicting the appearance of new pandemic and epidemic strains, as well as to the low cross–reactivity of inactivated and subunit vaccines, which are effective only against the strain of virus they were manufactured from. Vaccines which would provide a broad range of protection from potentially dangerous influenza virus strains are still a goal that is hard to achieve.

It is known that influenza infection can confer heterosubtypic immunity based on both the humoral and cellular immune responses. A heterosubtypic immune response can considerably decrease the duration of the disease and reduce the symptoms of infection by a different viral strain [21]. The induction of a heterosubtypic immune response occurs, among other things, due to the presence of conformational epitopes in hemagglutinin, which are conserved in various strains of the influenza virus.

**Fig. 6:**
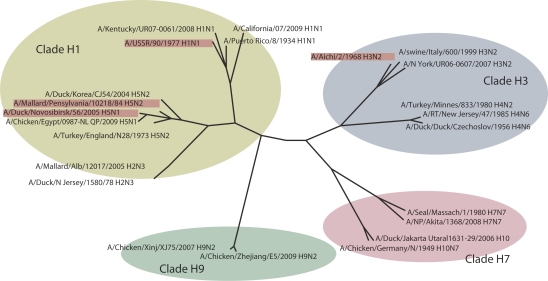
A phylogenetic tree of the amino acid sequences of hemagglutinins of the influenza A viruses of various subtypes. The various subtypes are divided into four clades based on their degree of phylogenetic relatedness. The strains used in this study (highlighted by color) belong to clades Н1 and Н3

The existence of a conformational epitope shared between several subtypes has been demonstrated for influenza A hemagglutinin [10]. In recent studies, various subtypes of the influenza virus have been divided into groups (clades) according to the presence of conservative hemagglutinin epitopes, which are recognized by antibodies that thus are able to neutralize a wide range of viral subtypes. This can be explained by the genetic relations between hemagglutinins from different viral subtypes. Figure 6 shows a phylogenetic tree of the amino acid sequences of influenza virus A hemagglutinins for various subtypes. The Н 1, H2, and H5 subtypes are all related and thus have common conformational epitopes, while Н 9, H7, and H3 virus subtypes have a low degree of homology and thus do not exhibit cross–immunity among themselves. Since the first clade includes potentially pandemic viral subtypes ( Н 5, Н 1, and Н 2), this makes broad–specificity vaccines against this clade especially valuable [12]. Thus, vaccines that preserve the stable ternary structure of surface antigens should provide effective protection against various influenza virus strains. The preparation of subunit and inactivated vaccines can often disrupt the structure of viral antigens. The use of live influenza vaccines does yield antibodies with a broad specificity, but this method has its own significant drawbacks.

Immunization by recombinant adenoviral vectors which bear the surface protein genes of the influenza virus leads to the expression of influenza virus antigens on the surface of the cell without disrupting their ternary structure, which in turn makes the induction of a heterosubtypic immune response, including the production of broad–specificity antibodies recognizing conservative hemagglutinin epitopes, possible. This study used adenoviral vectors with deleted Е 1 and Е 3 genomic regions, which allowed us to obtain replication–defective adenoviral particles and gave the potential possibility to use them as vaccine vectors. The advantages of using recombinant adenoviruses are the high level of transgene expression in a wide range of eukaryotic host cells, the induction of both humoral and cellular responses to the transgene, and their safety for humans (as tested on volunteers) [16]. The first phase of clinical trials for a nasal vaccine, based on a recombinant replication–defective human adenovirus of the fifth serotype bearing a hemaglutinin gene from the H5 influenza virus, was successfully performed in 2008 in the United States [13].

Our study used hemagglutinin genes from the avian influenza viruses H5N1 and H5N2, since avian influenza viruses, H5N1 especially, are a cause of increasing anxiety. This virus is characterized by a mortality rate in excess of 50%, and, if it were to acquire the ability to spread from human to human, it would lead to a pandemic and cause a lot of casualties throughout the world. We have constructed adenoviral vectors Ad–HA5–1 and Ad–HA5–2 based on a serotype 5 human adenovirus, which contain hemagglutinin genes from viral strains A/Duck/Novosibirsk/56/2005 (H5N1) and A/Mallard/Pennsylvania/10218/84 (H5N2). An analysis of the amino acid sequence of these hemaglutinins shows a homology of 94.6% (http://align.genome.jp/). An analysis of the immunogenicity of the obtained Ad–HA5–2 adenovirus showed that a double instranasal injection of a recombinant virus expressing the H5N2 viral hemagglutinin gene induced the production of high titers of antibodies specific towards the avian influenza virus A/Mallard/Pennsylvania/10218/84 (H5N2).

We have also demonstrated that a double injection of the Ad–HA5–1 virus expressing the H5N1 viral hemagglutinin gene induced the production of high titers or cross–reactive antibodies against the avian influenza virus A/Mallard/Pennsylvania/10218/84 (H5N2).

Immunizing mice with Ad–HA5–2 and Ad–HA5–1 adenoviruses protected the animals from a highly lethal dose (50 LD_50_) of the A/Mallard/Pennsylvania/10218/84 (H5N2) influenza virus. A control immunization of mice by the Ad–null vector, which did not carry any gene expression cassette, allowed us to establish the role of the vector itself in the protection against influenza A viruses. It turned out that Ad–null immunization protected 20% of the animals from the influenza virus. This seems to be caused by the induction of a nonspecific antiviral immune response initiated by the injection of an adenoviral vector in the animal organism. Thus, we have demonstrated that recombinant adenoviruses, which bear the influenza virus H5N2 hemaggliutinin gene, can induce geterosubtypic immunity between H5N1 and H5N2 influenza virus subtypes.

**Fig. 7:**
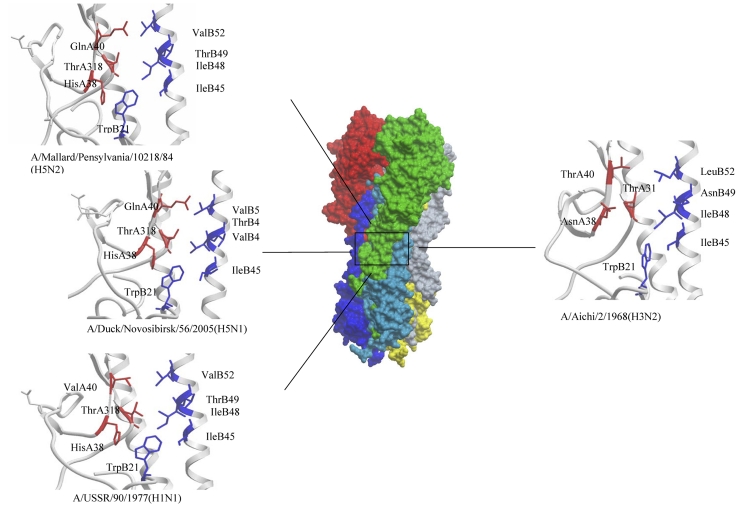
Conserved conformational epitopes of hemagglutinins recognized by neutralizing antibodies specific to the influenza strains used in this work and belonging to caldes Н1 and Н3. The surface of the hemagglutinin trimer is depicted in the middle; the HA1 chains are depicted in red, green and grey; and the HA2 chains are shown in dark blue, light blue and yellow. The region which contains the epitopes for the neutralizing antibodies is marked by a black box. The epitopes and the key amino acids that form them are depicted in HA1 (red) and HA2 (blue) hemagglutinin chains of various influenza viruses: A/Aichi/2/68(H3N2) (1EO8), A/USSR/90/77(H1N1) (1RVX), A/Mallard/Pensylvania/10218/84H5N2(H5N2) (1JSM) and A/Duck/Novosibirsk/56/2005(H5N1) (2IBX)

In order to verify the hypothesis that recombinant adenoviruses can induce broad–specificity antibodies that can neutralize viruses of different subtypes belonging to the H1 clade (H1, H2, H5, H6, H11, H13 and H16), we used the A/USSR/90/77 (H1N1) virus, which belonged to this clade, and A/Aichi/2/68 (H3N2), which did not. According to a study by M. Throsby and his colleagues [14], H5 and H1 virus hemagglutinins have a common conservative epitope which is formed by the amino acid residues His38, Gln40, and Thr318 in the HA1 subunit of hemagglutinin and Ile45, Ile48, Thr49, and Val52 in the НА 2 subunit. The hemaglutinins from Н 3 viruses have Asn and Thr in the 38th and 40th positions in the HA1 subunit, as opposed to His and Gln, and Thr and Val in positions 49 and 52 instead of Asn and Leu, respectively. An analysis of the amino acid sequences of influenza virus hemagglutinins in strains A/USSR/90/77 (H1N1), A/ Mallard/Pennsylvania/10218/84 (H5N2), A/duck/Novosibirsk/56/2005 (H5N1), and A/Aichi/2/68 (H3N2), which were all used in this study, showed that the described hemagglutinin epitopes are highly homologues in subtypes H1 and H5. Only the strain A/duck/Novosibirsk/56/2005 (H5N1) has a single substitution (the Gln in position 40 is substituted for Val) (Fig. 7). This epitope is, however, significantly different in strain A/Aichi/2/68 (H3N2), which does not belong to the first clade of influenza viruses. It was shown that double intranasal immunization with Ad–HA5–2 induced the production of antibodies specific to the A/USSR/90/77 (H1N1) virus strain. The titer of antibodies that inhibited the agglutination of erythrocytes by the H1N1 virus in sera extracted from immunized mice was significantly higher than that in the control group sera. On the other hand, the antibody titers against the A/Aichi/2/68 (H3N2) virus as assayed by hemagglutination–inhibition reaction in immunized mice were statistically insignificant when compared with the control for nonspecific inhibition by serum components.|

In order to assay the protective characteristics of the recombinant Ad–HA5–2 adenovirus against lethal doses (10 LD_50_) of A/USSR/90/77 (H1N1) and A/Aichi/2/68(H3N2) viruses, we performed a double intranasal immunization of mice. We demonstrated that immunization by a recombinant adenovirus conferred 100% protection against a 10 LD_50_ dose of the influenza virus A/USSR/90/77 (H1N1) and, on the other hand, protected only 20% of the mice from a 10 LD_50_ dose of the A/Aichi/2/68 (H3N2) virus, which probably indicates that this moderate protection was due to the nonspecific antiviral immune response induced by the injection of an adenoviral vector.

## CONCLUSIONS

This study is the first to demonstrate that the introduction of an influenza virus hemagglutinin gene via an adenoviral vector into animal cells induces a protective heterosubtypic cross–immune response not only against the viruses of the same subtype as used for the immunization, but also against viruses from various subtypes which belong to the same clade.

These data allow us to conclude that adenoviral vectors can work as a universal basis for the production of vaccines against both seasonal and pandemic influenza virus strains.
